# Supplementary material for: Non-Communicable Disease Burden and Dietary Determinants in Women of Reproductive Age in Sub-Saharan Africa: A Scoping Review
Source: Diseases. 2025 Sep 24;13(10):313. doi: 10.3390/diseases13100313 (PMC12564497; doi:10.3390/diseases13100313)
Supplement: Supplementary file 1 [file diseases-13-00313-s001.zip › Modjadji Scoping R Supplementary File S2.pdf]

Supplementary File S2

Table S1: Search strategy adapted on databases.

Database	Search	Reco
**PubMed**	Fields] OR ""hypertension s""[All Fields] OR ""hypertensions""[All Fiel ""hypertensive""[All Fields] OR ""hypertensive s""[All Fields ""hypertensives""[All Fields]) OR ""overweight obesity""[All Field (""dyslipidaemias""[All Fields] OR ""dyslipidemias""[MeSH Terms ""dyslipidemias""[All Fields] OR ""dyslipidaemia""[All Fields] ""dyslipidemia""[All Fields]) OR (""diabetes mellitus""[MeSH Term (""diabetes""[All Fields] AND ""mellitus""[All Fields]) OR ""diabetes mellit Fields]) OR (""hyperglycemia""[MeSH Terms] OR ""hyperglycemia""[All Fie (""high""[All Fields] AND ""blood""[All Fields] AND ""sugar""[All Fields]) OI blood sugar""[All Fields]) OR (""hypertension""[MeSH Terms ""hypertension""[All Fields] OR (""high""[All Fields] AND ""blood""[All Field ""pressure""[All Fields]) OR ""high blood pressure""[All Fields]) OR (""cardio diseases""[MeSH Terms] OR (""cardiovascular""[All Fields] AND ""diseas Fields]) OR ""cardiovascular diseases""[All Fields])) AND (((""diet""[MeSH Te ""diet""[All Fields] OR ""dietary""[All Fields] OR ""dietaries""[All Fields] (""analysis""[MeSH Subheading] OR ""analysis""[All Fields] OR ""determinati Fields] OR ""determinant""[All Fields] OR ""determinants""[All Field ""determinate""[All Fields] OR ""determinated""[All Fields] OR ""determinat Fields] OR ""determinating""[All Fields] OR ""determinations""[All Fiel ""determine""[All Fields] OR ""determined""[All Fields] OR ""determines""[Al OR ""determining""[All Fields])) OR ((""diet""[MeSH Terms] OR ""diet""[All OR ""dietary""[All Fields] OR ""dietaries""[All Fields]) AND (""factor""[All Fie ""factor s""[All Fields] OR ""factors""[All Fields])) OR (""dietary patterns"" Terms] OR (""dietary""[All Fields] AND ""patterns""[All Fields]) OR "" patterns""[All Fields]) OR (""nutritional status""[MeSH Terms] OR (""nutrition Fields] AND ""status""[All Fields]) OR ""nutritional status""[All Field ((""diet""[MeSH Terms] OR ""diet""[All Fields] OR ""dietary""[All Fiel ""dietaries""[All Fields]) AND (""diverse""[All Fields] OR ""diversely""[All Fie ""diversities""[All Fields] OR ""diversity""[All Fields])) OR (""eating""[MeSH OR ""eating""[All Fields] OR (""nutrient""[All Fields] AND ""intake""[All Fiel ""nutrient intake""[All Fields]) OR ((""family characteristics""[MeSH Terr (""family""[All Fields] AND ""characteristics""[All Fields]) OR " characteristics""[All Fields] OR ""household""[All Fields] OR ""househol Fields] OR ""household s""[All Fields] OR ""householder""[All Field ""householder s""[All Fields] OR ""householders""[All Fields]) AND security""[MeSH Terms] OR (""food""[All Fields] AND ""security""[All Fiel ""food security""[All Fields])) OR (""food insecurity""[MeSH Terms] OR (""fo Fields] AND ""insecurity""[All Fields]) OR ""food insecurity""[All Field (""eating""[MeSH Terms] OR ""eating""[All Fields] OR (""nutritional""[All Field ""intake""[All Fields]) OR ""nutritional intake""[All Fields]) OR (""eating"" Terms] OR ""eating""[All Fields] OR (""dietary""[All Fields] AND ""inta	11

	Fields]) OR ""dietary intake""[All Fields]))) AND (""africa, southern""[MeSH OR (""africa""[All Fields] AND ""southern""[All Fields]) OR ""southern afri Fields] OR (""southern""[All Fields] AND ""africa""[All Fields]) OR (africa""[MeSH Terms] OR (""south""[All Fields] AND ""africa""[All Fields]) OR africa""[All Fields]) OR (""africa south of the sahara""[MeSH Terms] OR (""afri Fields] AND ""south""[All Fields] AND ""sahara""[All Fields]) OR ""africa sout sahara""[All Fields] OR (""sub""[All Fields] AND ""saharan""[All Fields ""africa""[All Fields]) OR ""sub saharan africa""[All Fields]) OR (""africa"" Terms] OR ""africa""[All Fields] OR ""africa s""[All Fields] OR ""afric Fields]))","2,165",07:47:21	
**Scopus**	(TITLE-ABS-KEY("Reproductive-Aged Women" OR "Childbearing Wom "Women 15-49 Years" OR "Women of Childbearing Years" OR "Wo Reproductive Age" OR WRA OR women OR female))<br>AND<br>(TITLE-ABS-KEY("Non-communicable Diseases" OR diabetes OR "insulin res OR hypertension OR overweight OR obesity OR dyslipidemia OR "diabetes n OR "high blood sugar" OR "high blood pressure" OR "cardiovascular diseases<br>AND<br>(TITLE-ABS-KEY("Dietary Determinants" OR "Dietary Factors" OR "Dietary P OR "Nutritional Status" OR "Dietary Diversity" OR "Nutrient Intake" OR "Ho Food Security" OR "Food Insecurity" OR "nutritional intake" OR "dietary intal<br>AND<br>(TITLE-ABS-KEY("Southern Africa" OR "South Africa" OR "sub-Saharan Afr Africa))	45
**Google sc**	("reproductive-aged women" OR "childbearing women" OR "women 15-49 ye "women of childbearing years" OR "women in reproductive age" OR WRA OR OR female)<br>AND<br>("non-communicable diseases" OR diabetes OR "insulin resistance" OR hyper OR overweight OR obesity OR dyslipidemia OR "diabetes mellitus" OR "hig sugar" OR "high blood pressure" OR "cardiovascular diseases")<br>AND<br>("dietary determinants" OR "dietary factors" OR "dietary patterns" OR "nu status" OR "dietary diversity" OR "nutrient intake" OR "household food secu "food insecurity" OR "nutritional intake" OR "dietary intake")<br>AND<br>("southern Africa" OR "South Africa" OR "sub-Saharan Africa" OR Africa)	11
